# Wilms Tumor Treatment Outcomes: Perspectives From a Low-Income Setting

**DOI:** 10.1200/JGO.2016.005389

**Published:** 2016-12-21

**Authors:** Festus Njuguna, Hugo A. Martijn, Robert Tenge Kuremu, Peter Saula, Patel Kirtika, Gilbert Olbara, Sandra Langat, Steve Martin, Jodi Skiles, Terry Vik, Gertjan J.L. Kaspers, Saskia Mostert

**Affiliations:** **Festus Njuguna**, **Robert Tenge Kuremu**, **Peter Saula**, **Patel Kirtika**, **Gilbert Olbara**, and **Sandra Langat,** Moi University, Eldoret, Kenya; **Hugo A. Mattijn**, **Gertjan J.L. Kaspers**, and **Saskia Mostert**, Vrije Universiteit Medical Center, Amsterdam, the Netherlands; and **Steve Martin**, **Jodi Skiles**, and **Terry Vik**, Indiana University School of Medicine, Indianapolis, IN.

## Abstract

**Purpose:**

Wilms tumor is the commonest renal malignancy in childhood. Survival in high-income countries is approximately 90%, whereas in low-income countries, it is less than 50%. This study assessed treatment outcomes of patients with Wilms tumor at a Kenyan academic hospital.

**Patients and Methods:**

We conducted a retrospective medical record review of all children diagnosed with Wilms tumor between 2010 and 2012. Data on treatment outcomes and various sociodemographic and clinical characteristics were collected.

**Results:**

Of the 39 patients with Wilms tumor, 41% had event-free survival, 31% abandoned treatment, 23% died, and 5% had progressive or relapsed disease. Most patients presented at an advanced stage: stage I (0%), II (7%), III (43%), IV (40%), or V (10%). The most likely treatment outcome in patients with low-stage (I to III) disease was event-free survival (67%), whereas in those with high-stage (IV to V) disease, it was death (40%). No deaths or instances of progressive or relapsed disease were recorded among patients with low-stage disease; their only reason for treatment failure was abandonment of treatment. Stage of disease significantly affected treatment outcomes (*P* = .014) and event-free survival estimates (*P* < .001). Age at diagnosis, sex, duration of symptoms, distance to hospital, and health insurance status did not statistically significantly influence treatment outcomes or event-free survival estimates.

**Conclusion:**

Survival of patients with Wilms tumor in Kenya is lower compared with that in high-income countries. Treatment abandonment is the most common cause of treatment failure. Stage of disease at diagnosis statistically significantly affects treatment outcomes and survival.

## INTRODUCTION

Wilms tumor is the most common primary renal malignancy in children. It accounts for 5% of childhood malignancies.^1^ It is thought to arise from nephrogenic rests, which are foci of persistent metanephrenic cells.^2^ Survival rates have improved from 20% in the 1960s to approximately 90% currently in high-income countries; middle-income countries have survival rates of approximately 80%.^2,3^ This has been achieved through cooperative study groups as well as use of multimodal approaches to therapy. The two main study groups that have been involved are the National Wilms’ Tumor Study Group and the International Society of Pediatric Oncology (SIOP).^2,4,5^

Low-income countries, however, have survival rates between 20% and 50%.^1-3^ Reasons for the low survival in low-income countries include limited access to proper medical care as a result of lack of facilities for treatment, shortage of personnel, long distances to treatment centers, poor infrastructure, and limited public transport facilities. These factors lead to late presentation, which also affects outcomes. Other contributors to the low survival include lack of health insurance, abandonment of treatment, and lack of a multidisciplinary approach to the management of patients. Treatment includes surgery and chemotherapy, as well as radiotherapy for metastatic disease.^2,3,5^

The aims of our study were to assess the treatment outcomes of children presenting with Wilms tumor at a Kenyan academic hospital and to evaluate the influence of various sociodemographic and clinical characteristics (eg, age at diagnosis, sex, duration of symptoms, stage of disease, distance to hospital, and health insurance status) on treatment outcomes.

## PATIENTS AND METHODS

### Setting

Kenya is situated in East Africa and is a low-income country with a population of approximately 43 million people.^6^ Most of the population (45%) lives below the poverty line.^7^ This study was carried out at Moi Teaching and Referral Hospital (MTRH), which is an academic hospital in Eldoret, a town 300 km northwest of the capital city Nairobi. The hospital has a capacity of approximately 800 beds, including 72 beds in the pediatric ward, of which 12 are dedicated to pediatric oncology.^8^ Approximately 120 pediatric oncology patients are seen in the hospital every year, in contrast to the expected number of 700 patients.^8^ One pediatrician is involved in the care of oncology patients. Two pediatric surgeons are involved in the surgical aspects of care. There is no radiotherapy facility in Eldoret; patients who require radiotherapy are referred to a center in Nairobi. Families pay for their hospital bills through health insurance or out of pocket. However, only approximately 10% of the Kenyan population have health insurance, which is provided by the government-owned and -controlled National Hospital Insurance Fund (NHIF) or through private insurance companies. Kenyan citizens can enroll with NHIF and pay a set monthly fee. Payments are dependent on level of income for those who are formally employed, whereas those who are self-employed or casual workers pay a monthly fee of approximately US$12. NHIF provides cover for inpatient care for the entire family in government-owned health facilities.^9,10^

Patients with Wilms tumor are treated according to a protocol modeled on the SIOP approach. Treatment is started after imaging via computed tomography confirms an intrarenal tumor. All patients receive 6 weeks of preoperative chemotherapy with vincristine, dactinomycin, and doxorubicin. Vincristine is administered once per week; dactinomycin is administered in weeks 1, 3, and 5; and doxorubicin is administered in weeks 1 and 5 only. Patients are then scheduled for surgery in week 7 or 8 of treatment. Disease staging is performed intraoperatively, using imaging to detect lung or liver metastases. Staging guides the decision on postoperative treatment. Postoperatively, patients with stage I disease receive 4 weeks of vincristine and dactinomycin. Children with stage II or III disease receive 16 weeks of vincristine and dactinomycin; those with stage III disease are referred for radiotherapy as well. Children with stage IV disease, as well as those with anaplastic histology regardless of stage, receive vincristine, dactinomycin, and doxorubicin for 16 weeks. Patients with stage V disease receive the same preoperative chemotherapy outlined here; the decision on further treatment depends on preoperative imaging and findings at surgery.

### Study Design

This was a retrospective medical record study. All children presenting with Wilms tumor at MTRH between January 1, 2010, and December 31, 2012, age between 0 and 16 years at diagnosis were included. It is important to note that we did not select patients for our analysis; rather, we included all patients who were diagnosed with Wilms tumor.

The names and inpatient numbers of patients diagnosed with Wilms tumor were extracted from the pediatric oncology database. Files were obtained from the medical record department. Sociodemographic and clinical characteristics were extracted from patients’ medical records using a data collection form.

Sociodemographic characteristics included age at diagnosis, sex, ethnicity, patient residence, and enrollment in NHIF. A patient’s residence was used to determine the distance from MTRH, which was subsequently categorized into distance of 100 km or less or more than 100 km.

Clinical characteristics included date of diagnosis, disease stage, time to event, and treatment outcome. Disease stage was determined using imaging to detect any lung or liver metastases, as well as through the information derived from intraoperative findings. For further analysis on outcomes, we grouped those with nonmetastatic stage I to III disease into low-stage and those with stage IV or V disease into high-stage groups. Treatment outcomes were classified as abandonment of treatment, death, progressive or relapsed disease, and event-free survival. Abandonment of treatment was defined as either not starting or not continuing planned treatment during 4 or more sequential weeks.^11^

### Data Analysis

Data analysis and management were performed using SPSS software (version 20; SPSS, Chicago, IL). Frequency distributions, means, and medians were calculated. The relationship between treatment outcomes and sociodemographic or clinical characteristics was evaluated using χ^2^ and Fisher’s exact tests. The probability of event-free survival was estimated using the Kaplan-Meier method; estimates were compared using the log-rank test. Event-free survival was measured from date of Wilms tumor diagnosis to first treatment failure or date of last follow-up. Treatment failure included abandonment of treatment, death, and progressive or relapsed disease.

## RESULTS

A total of 39 patients with Wilms tumor presented to the hospital during the study period. Girls comprised 52% of patients. Table 1 lists sociodemographic and clinical characteristics. Almost all patients (97%) were referred to MTRH from other health facilities. A majority (91%) were referred from secondary-level public health facilities, whereas the rest were referred by private clinics (3%), private hospitals (3%), or tertiary-level hospitals (3%). Before patients presented to MTRH, only 16% had received a possible diagnosis of Wilms tumor, and none had received any treatment specifically for Wilms tumor. A majority of patients presented at later stages. There was no patient with stage I disease. Children were diagnosed with: stage II (7%), III (43%), IV (40%), or V (10%) disease. Of the 39 patients, 54% lived more than 100 km from MTRH. At time of diagnosis, 39% of patients had NHIF. Of those who did not have NHIF at diagnosis, most (83%) registered while undergoing treatment at MTRH, bringing the total enrollment level to 90%.

**Table 1:**
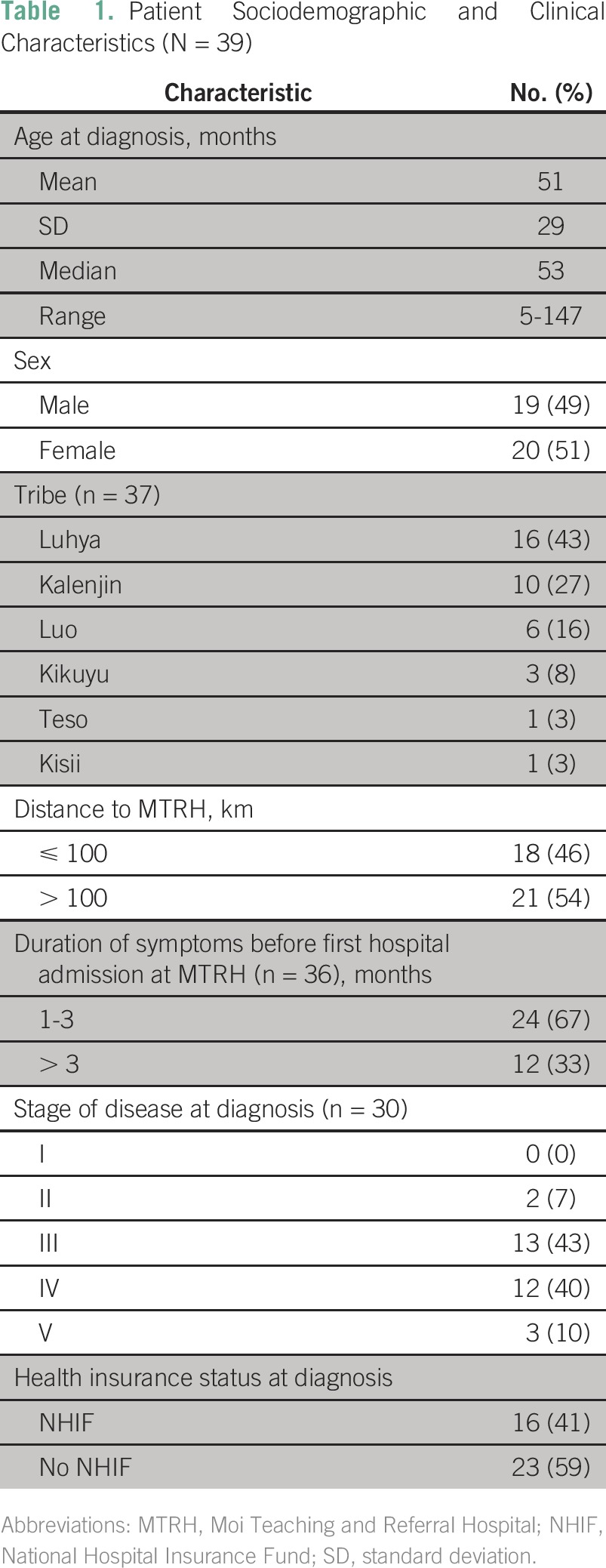
Patient Sociodemographic and Clinical Characteristics (N = 39)

**Fig 1:**
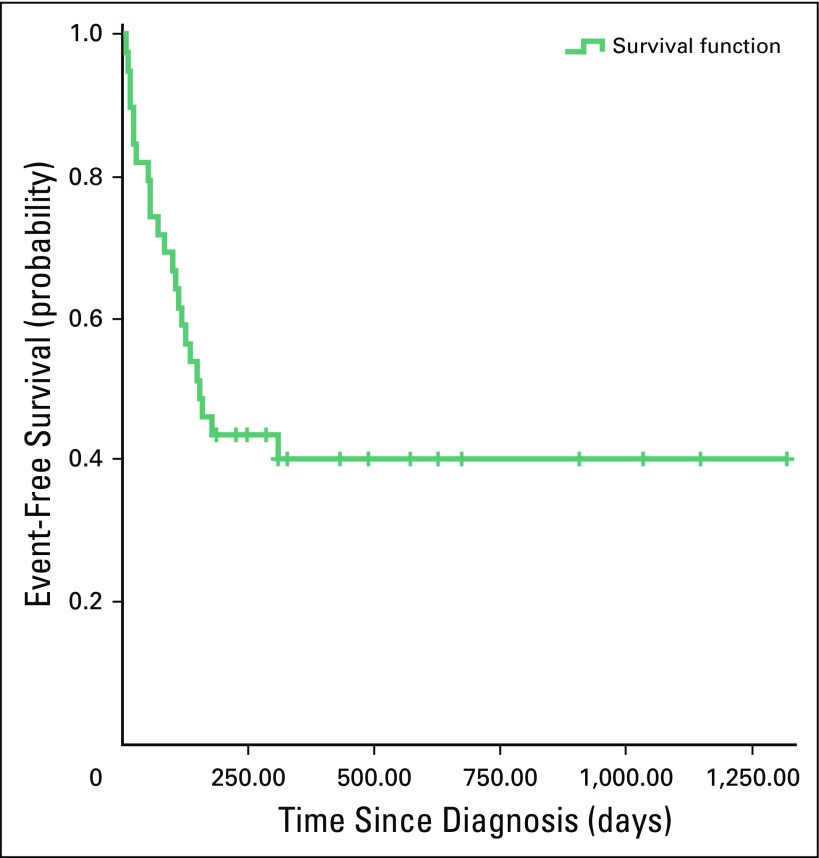
Kaplan-Meier estimates of event-free survival in children with Wilms tumor (N = 39). Events included abandonment of treatment, death, and progressive or relapsed disease. Crosses indicate censored patients.

The overall 3-year survival rate was 41%. Figure 1 shows the event-free survival estimate of all children with Wilms tumor.

As summarized in Table 2, the most common cause of treatment failure was abandonment of treatment (31%), and the second most common was death (23%). All deaths occurred within 4 months of diagnosis, with 78% of these children dying within the first 2 months. The least common cause of treatment failure was progressive or relapsed disease (5%).

**Table 2:**
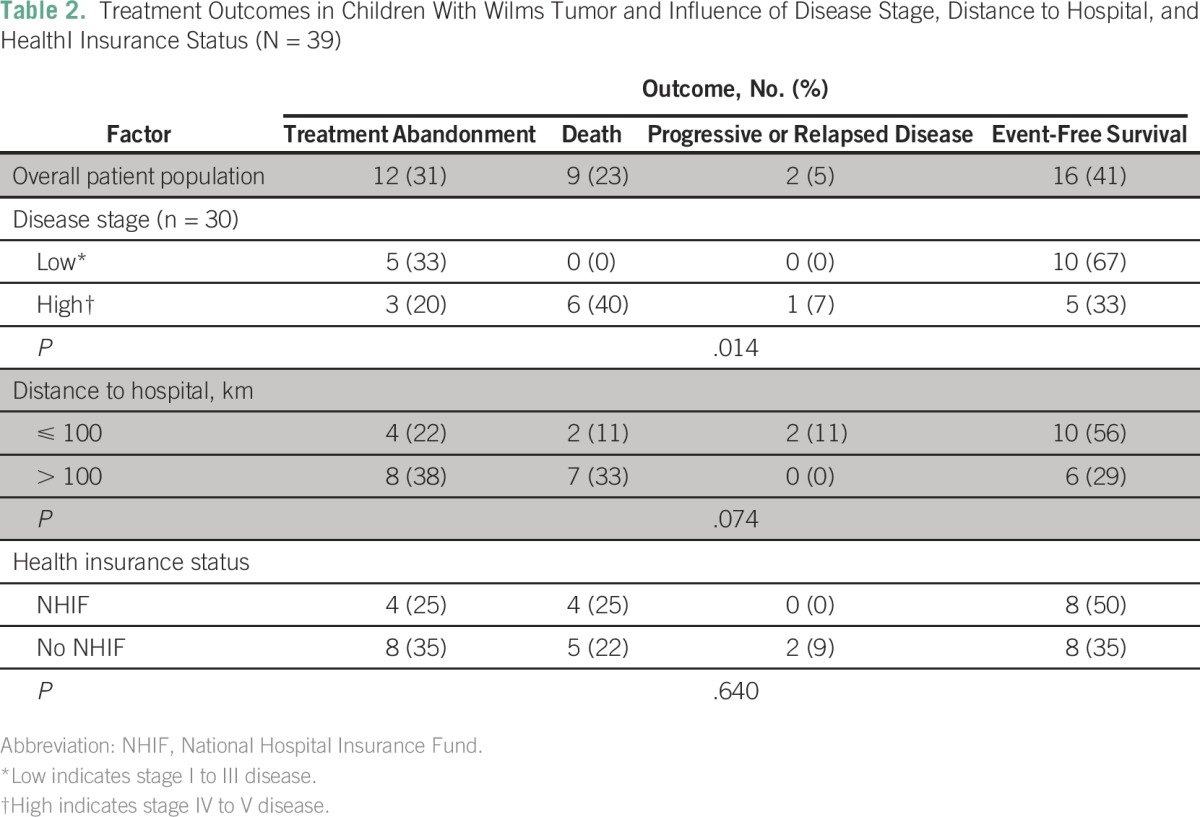
Treatment Outcomes in Children With Wilms Tumor and Influence of Disease Stage, Distance to Hospital, and HealthI Insurance Status (N = 39)

**Fig 2:**
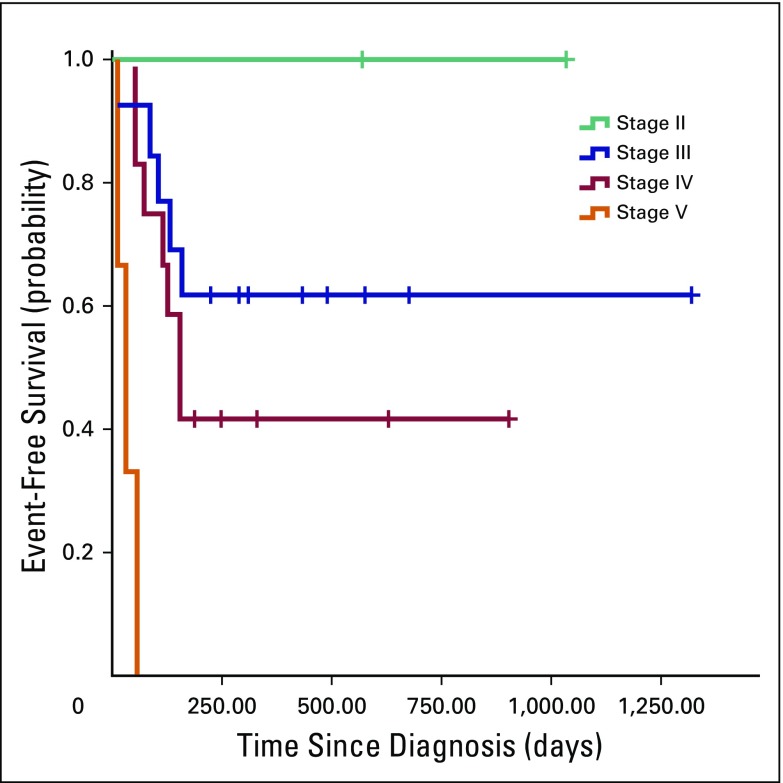
Kaplan-Meier estimates of event-free survival in children with Wilms tumor per disease stage at diagnosis (*P* < .001). Events included abandonment of treatment, death, and progressive or relapsed disease. Crosses indicate censored patients.

Of 30 patients with documented stage of disease, 50% had low-stage (I to III) and 50% had high-stage (IV to V) disease. The most likely treatment outcome in patients with low-stage disease was event-free survival (67%), whereas in patients with high-stage disease, it was death (40%). No deaths or instances of progressive or relapsed disease occurred among patients with low-stage disease. As summarized in Table 2, differences in treatment outcomes between children with low- and high-stage disease were significant (*P* = .014). Figure 2 shows that event-free survival estimates differed significantly between patients with stage II, III, IV and V disease (*P* < .001).

**Fig 3:**
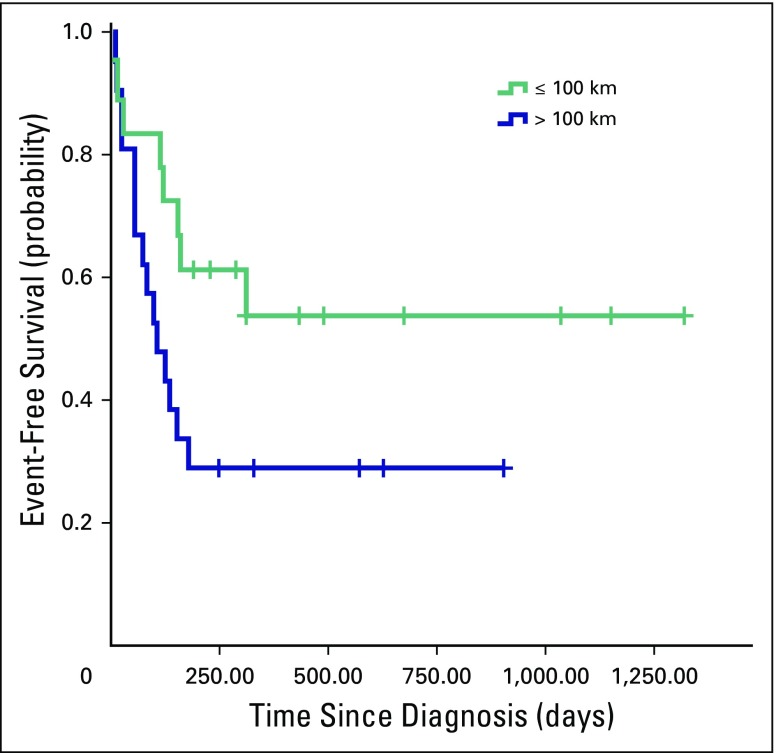
Kaplan-Meier estimates of event-free survival in children with Wilms tumor per distance from hospital (*P* = .063) Events included abandonment of treatment, death and progressive or relapsed disease. Crosses indicate censored patients.

**Fig 4:**
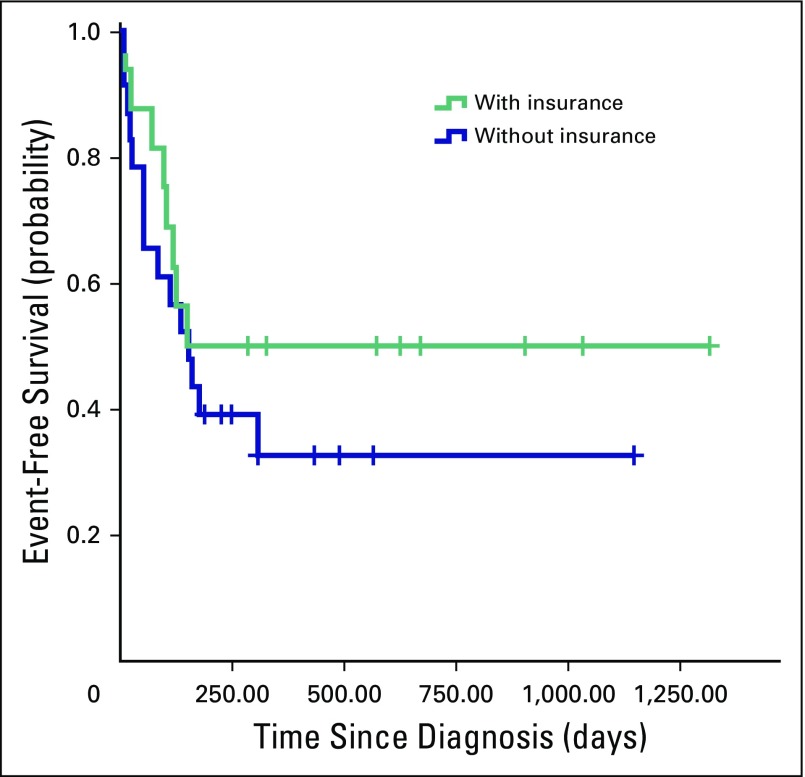
Kaplan-Meier estimates of event-free survival in children with Wilms tumor per National Hospital Insurance Fund health insurance status at diagnosis (*P* = .358). Events included abandonment of treatment, death, and progressive or relapsed disease. Crosses indicate censored patients.

Other sociodemographic and clinical characteristics (ie, age at diagnosis, sex, duration of symptoms, distance to hospital, and health insurance status) did not have a statistically significant influence on treatment outcomes or event-free survival estimates. Figures 3 and 4 illustrate that living at a shorter distance from MTRH and having health insurance at diagnosis led to better chances of survival, but this did not reach statistical significance (*P* = .063 and .358, respectively).

## DISCUSSION

This study demonstrated a survival rate of 41% among patients diagnosed with Wilms tumor at MTRH between the years 2010 to 2012. This is a great improvement from the survival rate of 29% that was documented for those patients treated at the institution between the years 2000 and 2007.^12^ This improvement may be attributed to several factors. The hospital adopted the SIOP approach to the management of Wilms tumor during the timeframe of our study. In the previous study, some patients never received any preoperative chemotherapy, and mortality was high. In 2009, the hospital developed a protocol manual that was used to manage all patients with cancer. Use of protocols and establishment of a multidisciplinary team have been demonstrated to lead to better outcomes. We now have competent pediatric surgeons, psychological counselors, social workers, and pharmacists involved in the care of patients with Wilms tumor. A team of dedicated pediatric oncology nurses cares for the children, unlike in the past, when nurses were moved from the department every few months. This has increased nurses’ knowledge and experience, which has resulted in better patient care. Supportive care has also improved over time through use of a protocol for management of febrile neutropenia and better availability of antibiotics. Nutritional care has improved significantly. Previously, cultural beliefs and associations with death prevented both the medical team and the families from using nasogastric feeding. Now most children do undergo nasogastric tube feeding, which allows feeding even when children have decreased appetite or mucositis. All patients were also actively encouraged to register with NHIF and were provided with assistance whenever possible. All these interventions have been achieved through collaboration with two partners in high-income countries: the Indiana University School of Medicine in the United States and the Vrije Universiteit Medical Center in the Netherlands. This collaboration has led to the transfer of knowledge among physicians, nurses, and other support staff, achieved through exchange visits, teleconferences in which patient care is discussed, and training workshops held in Eldoret every year.

High-income countries have reported high survival rates among children with Wilms tumor. In the United Kingdom, an overall survival rate of 88% was documented on 10-year follow-up.^13^ Middle-income countries also have good survival rates, with China reporting a survival rate of 81%.^14^ However, the survival rates are still low in low-income countries, especially in Africa. A 2-year survival rate of 25% was reported from an eight-center Wilms tumor treatment collaborative effort in Africa.^15^ In Malawi, the survival rate is 46%.^16^ These low survival rates have been attributed to several factors, including high treatment abandonment and treatment-related mortality.^17^

The rate of treatment abandonment was 31% in our study. This is a decrease from the 54% abandonment rate that we reported before in the same hospital for the period of 2007 to 2009, although the latter study examined all types of cancer.^18^ This figure is also lower than the 42% reported previously among patients with Wilms tumor at the same institution.^12^ In a study that examined several hospitals in Kenya, a 50% rate of patients lost to follow-up was reported among those with Wilms’ tumor, although this included both patients who abandoned therapy as well as patients who were lost to follow-up after finishing treatment.^19^ In Africa, Wilms tumor treatment abandonment rates vary between 14% and 48%.^15^ Abandonment in this setting is attributed to a lack of parental education on Wilms tumor by medical staff, parents’ misunderstanding of treatment protocols, and families’ financial difficulties.^20^ Abandonment of therapy contributes to a large extent to poor outcomes in pediatric oncology in low-income countries. In our study, abandonment was the only adverse outcome among those patients with stage II or III disease. If this phenomenon is addressed adequately, survival in this group could improve, approaching that reported in high-income countries.

A majority of patients of our study presented with late-stage disease. Those who had stage II disease had good outcomes, in contrast to those with later stages of disease. A multicenter study of Wilms tumor involving French-speaking countries in Africa reported that patients with stage III or IV disease comprised 41% of all patient cases.^17^ In South Africa, those with stage III or IV disease comprised 49% of patient cases.^21^ In both these studies, patients with stage V disease were excluded from analysis. This indicates that late presentation is still a major issue in low-income settings. It could possibly be explained by circumstances that lead to both patient and health care system delays. Patient delays usually result from outdated health beliefs, poor reputation of public hospitals, preference for alternative medicine, and financial difficulties coupled with lack of health insurance. Health care system delays result from unavailability of the qualified personnel or equipment required to make correct diagnoses.^22,23^

Disease stage has been documented as one of the most important prognostic factors. However, there are still huge differences when we compare outcomes in high- versus low-income countries. In the United Kingdom, an overall survival rate of 81% for stage IV disease was reported.^13^ In Africa, in the French-speaking collaborative group, children with stage IV disease had an overall survival rate of 49%, and in South Africa, the survival rate was 57%.^15,21^ Disparities in survival between high- and low-income countries are worse in the more advanced disease stages; however, most patients from low-income countries present with advanced disease. Therefore, to improve outcomes, we should concentrate not only on improving the standards of care but also on diagnosing patients with early-stage disease. Increasing awareness of childhood cancer among health care workers is paramount. Having ultrasound machines as well as trained personnel in most primary care centers could lead to increased detection rates. This strategy could have the potential of increasing survival with less strain on the health care system.

Patients living more than 100 km from MTRH had lower chances of survival compared with those living nearer to the hospital, although this did not reach statistical significance. The most likely treatment outcome in patients within 100 km of MTRH was event-free survival, whereas in patients living farther from MTRH, it was abandonment of treatment. Distance and transport costs have been demonstrated to increase chances of abandonment and thereby decrease survival in pediatric oncology.^24,25^ In a previous study among families of children with cancer who abandoned treatment at MTRH, it was found that long distance to the hospital led to higher costs of transportation and affected the ability to keep appointments.^18^ Most Kenyan families use public transport to reach MTRH. However, Kenyan public transport is not well organized. The number and quality of roads are limited. There are no fixed routes, timetables, or fares.^18^ These infrastructural obstacles may ultimately affect the survival of children with Wilms tumor.

Although only 39% of families had health insurance before coming to MTRH, this number is higher than the national figure of 10%.^26^ Previous studies in the Kenyan setting have shown that having NHIF at diagnosis significantly decreases abandonment and improves childhood cancer survival.^18,20^ This taught our team that it is important to enroll patients in NHIF. In the pediatric oncology ward at MTRH, the physicians and nurses therefore now continually inform families about the need for NHIF. Particularly for children with potentially good prognoses, like those with Wilms tumor, medical staff make sure that families get NHIF. Support staff help families to collate all documents required for this purpose and direct them on which office to visit. Most families in our study subsequently enrolled in NHIF during hospitalization. This illustrates that if families are given the right information and are assisted in obtaining health insurance, many of them are willing to do so. The government should have mass media educational campaigns on the benefits and procedures of registering with NHIF.

The main limitations in this study were the small sample size and the fact that, because it was a retrospective medical record review, some data were missing. In conclusion, the survival rate of patients with Wilms tumor at MTRH improved between the years 2010 and 2012 as compared with 2000 to 2007. The main reason for treatment failure was abandonment of treatment. Disease stage at diagnosis significantly affected treatment outcomes and event-free survival estimates. Age at diagnosis, sex, duration of symptoms, distance to hospital, and having health insurance at diagnosis did not predict survival.

On the basis of the findings of our study, we acknowledge that abandonment of treatment needs to be addressed. Providing proper parental education and financial support would be useful strategies. To help reduce the number of children presenting with late-stage disease and improve access to conventional health care facilities, we recommend that the government initiate mandatory universal health insurance coverage. Health care workers should be trained on the clinical features of Wilms tumor. This should be done by incorporating training on childhood cancers into the curricula of medical training institutions, as well as through continuous professional development for those who have already graduated. To reduce transportation difficulties for families living far from the hospital, establishing satellite clinics and family guesthouses near the hospital could be beneficial. Ultimately, all these interventions could improve survival of children with Wilms tumor.
